# HCN4 ion channel function is required for early events that regulate anatomical left-right patterning in a nodal and lefty asymmetric gene expression-independent manner

**DOI:** 10.1242/bio.025957

**Published:** 2017-08-17

**Authors:** Vaibhav P. Pai, Valerie Willocq, Emily J. Pitcairn, Joan M. Lemire, Jean-François Paré, Nian-Qing Shi, Kelly A. McLaughlin, Michael Levin

**Affiliations:** 1 Allen Discovery Center at Tufts University, 200 Boston Ave, Suite 4600, Medford, MA 02155, USA; 2 Department of Medicine at University of Wisconsin-Madison, Madison, WI 53792, USA

**Keywords:** HCN4, Bioelectricity, Ion channels, Laterality, *Xenopus*

## Abstract

Laterality is a basic characteristic of all life forms, from single cell organisms to complex plants and animals. For many metazoans, consistent left-right asymmetric patterning is essential for the correct anatomy of internal organs, such as the heart, gut, and brain; disruption of left-right asymmetry patterning leads to an important class of birth defects in human patients. Laterality functions across multiple scales, where early embryonic, subcellular and chiral cytoskeletal events are coupled with asymmetric amplification mechanisms and gene regulatory networks leading to asymmetric physical forces that ultimately result in distinct left and right anatomical organ patterning. Recent studies have suggested the existence of multiple parallel pathways regulating organ asymmetry. Here, we show that an isoform of the hyperpolarization-activated cyclic nucleotide-gated (HCN) family of ion channels (hyperpolarization-activated cyclic nucleotide-gated channel 4, HCN4) is important for correct left-right patterning. HCN4 channels are present very early in *Xenopus* embryos. Blocking HCN channels (*I_h_* currents) with pharmacological inhibitors leads to errors in organ situs. This effect is only seen when HCN4 channels are blocked early (pre-stage 10) and not by a later block (post-stage 10). Injections of *HCN4-DN* (dominant-negative) mRNA induce left-right defects only when injected in both blastomeres no later than the 2-cell stage. Analysis of key asymmetric genes' expression showed that the sidedness of *Nodal*, *Lefty*, and *Pitx2* expression is largely unchanged by HCN4 blockade, despite the randomization of subsequent organ situs, although the area of *Pitx2* expression was significantly reduced. Together these data identify a novel, developmental role for HCN4 channels and reveal a new *Nodal-Lefty-Pitx2* asymmetric gene expression-independent mechanism upstream of organ positioning during embryonic left-right patterning.

## Abstract (precis)

**Summary:** We identify a novel, developmental role for HCN4 channels and reveal a new *Nodal-Lefty-Pitx2*-independent, non-canonical mechanism upstream of organ positioning during embryonic left-right patterning. This article has an associated First Person interview with the first author of the paper as part of the supplementary information.

## INTRODUCTION

Invariant left-right asymmetry is a fundamental aspect of all life, from single cell organisms to plants and animals with complex body plans like humans (Chen et al., 2012; Coutelis et al., 2008; Davison et al., 2016; Dimonte et al., 2016; Gros et al., 2009; Hashimoto, 2002; Kuroda et al., 2009; Naganathan et al., 2014; Petzoldt et al., 2012; Pohl, 2011; Spéder et al., 2007; Thitamadee et al., 2002; Wan et al., 2011; Xu et al., 2007; Yost, 1990, 1991). Consistent orientation of the left-right (LR) axis is a difficult problem for an embryo to solve in a universe that does not macroscopically distinguish left from right, and must be done reliably and accurately to achieve correct organization of internal organ structures. Errors in left-right asymmetry form a large and important class of human birth defects, affecting almost all major visceral organs, including the heart and the brain (Burn, 1991; Cohen et al., 2007; Hoffman and Kaplan, 2002; Peeters and Devriendt, 2006; Ramsdell, 2005). Hence, understanding the establishment of consistent laterality in the organization of body plans is a fundamental question in evolutionary and developmental biology, with important implications for addressing birth defects via regenerative medicine.

It is becoming clear that the origins of left-right asymmetry lie in physical aspects of cytoskeletal chirality (Naganathan et al., 2016; Suzuki et al., 2017; Tee et al., 2015; Wan et al., 2011), amplified immediately post-fertilization in the early embryos of many species (McDowell et al., 2016a,b; Okumura et al., 2008; Spéder et al., 2007; Vandenberg et al., 2013a; Vandenberg and Levin, 2013). The cytoskeletal chirality-mediated initiation of laterality is highly conserved across phyla, and even kingdoms (Levin and Nascone, 1997; Levin and Palmer, 2007; Lobikin et al., 2012; Okumura et al., 2008; Spéder et al., 2007). Multiple amplification and reinforcement mechanisms transmit this early left-right asymmetry across the entire developing embryo. These include asymmetric transport of ion translocators (Adams et al., 2006; Aw et al., 2008; Bessodes et al., 2012; Levin et al., 2002), charged molecules through gap junctions (Fukumoto et al., 2005a,b; Garic-Stankovic et al., 2008; Oviedo and Levin, 2007; Vandenberg et al., 2013b), and ciliary flow (Basu and Brueckner, 2008; Schweickert et al., 2007). Collectively, these signals trigger an embryo-wide asymmetric gene regulatory network, with a left-side expression of *Nodal-Lefty-Pitx2* as the primary node (Levin, 1998; Mercola and Levin, 2001; Nakamura and Hamada, 2012; Ramsdell and Yost, 1998; Raya and Izpisua Belmonte, 2004a,b). Finally, asymmetric generation of mechanical forces results in asymmetric organ structure and placement (Granados-Riveron and Brook, 2012; Voronov et al., 2004; Welsh et al., 2013).

Previous work has shown an important role of ion fluxes-mediated regulation of membrane voltage in determination of left-right laterality (Adams et al., 2006; Aw et al., 2008, 2010; Garic-Stankovic et al., 2008; Hibino et al., 2006; Levin et al., 2006, 2002; Morokuma et al., 2008; Oviedo and Levin, 2007; Shimeld and Levin, 2006). Like other mechanisms involved in left-right laterality determination, these also largely feed into the *Nodal-Lefty-Pitx2* node of gene regulatory networks. Recent evidence has uncovered a non-linearity between the cytoskeletal initiation of left-right asymmetry and establishment of organ situs, where errors in key aspects of the gene regulatory network, like the sidedness of *Nodal* expression, are bypassed to establish correct left-right organ situs despite randomized expression of upstream laterality determinant genes (Cota et al., 2006; McDowell et al., 2016a,b). These alternate pathways provide redundancy and robustness to the establishment of proper left-right organ situs, but are poorly understood. Here we report a new element of the endogenous bioelectric toolbox of left-right patterning, the hyperpolarization-activated cyclic nucleotide-gated channel 4 (HCN4), and present data suggesting that it mediates alternative pathways (bypassing the *Nodal-Lefty-Pitx2* cassette) involved in the establishment of left-right asymmetry in *Xenopus* embryos.

Hyperpolarization-activated cyclic nucleotide-gated (HCN) channels are a unique group of voltage-gated channels where the threshold voltage for opening of the channel is modulated by the metabolic state of the cell (levels of cyclic nucleotides like cAMP) (Biel et al., 2009; Wahl-Schott and Biel, 2009). They open at hyperpolarized membrane voltages (negative), giving rise to currents which are a mix of sodium and potassium ion fluxes. Among the four HCN isoforms (HCN1-4), HCN4 channels have been primarily studied in adult hearts as pacemaker channels (Scicchitano et al., 2012; Verkerk and Wilders, 2015), but recent evidence has demonstrated their presence in human and mouse embryonic cells (Cerbai et al., 1999; Qu et al., 2008; Robinson et al., 1997; Später et al., 2013; Vicente-Steijn et al., 2011; Yasui et al., 2001), and implicated them in cardiac patterning (Pitcairn et al., 2017). Because these channels have not been studied in the context of developmental bioelectricity (Adams and Levin, 2013; Levin, 2013, 2014a,b) or control of embryonic axial patterning, we characterized the role of HCN4 channels in embryonic left-right asymmetry establishment in *Xenopus* embryos.

Here we extend our recent work on HCN4 channels in cardiogenesis (Pitcairn et al., 2017) using a different set of misexpression conditions to target earlier events. We show that HCN4 channels are present in *Xenopus* embryos beginning at the first cleavage event (2-cell stage). Pharmacological inhibition of HCN channels (*I_h_* currents) by ZD7288 causes heterotaxia (randomization of organ situs) in *Xenopus* tadpoles, but only if embryos are exposed early (pre-stage 10, prior to the onset of gastrulation). Similarly, injections of mRNA encoding a dominant-negative protein (which blocks HCN4 channel function) randomize organ asymmetry if injected into both blastomeres at the 2-cell stage. Remarkably, despite the early period of action (prior to asymmetric gene expression), HCN4 channel disruption does not affect the laterality of subsequent *Nodal*, *Lefty*, or *Pitx2* expression, exhibiting significant latency in its effects on later organ situs. Together, these results are the first, to our knowledge, to show an early embryonic axial patterning role of this important ion channel, and implicate it in a pathway bypassing the asymmetric gene expression of *Nodal*, *Lefty*, and *Pitx2* in regulating left-right asymmetry.

## RESULTS

### Early exposure to pharmacological inhibitor ZD7288 induces heterotaxia

Our prior work demonstrated that interference with four different ion translocators (two channels and two pumps) induces heterotaxia, the independent randomization of organ positioning along the LR axis (Adams et al., 2006; Aw et al., 2008, 2010; Levin et al., 2002; Morokuma et al., 2008). To test whether HCN4 channels also play a role in embryonic left-right laterality determination, we first used the pharmacological inhibitor ZD7288 (100 µM), which blocks HCN channels (*I_h_* currents) via a trapping mechanism (Shin et al., 2001). To probe the timing of the endogenous role of HCN4 channels in development, we compared the results of exposures beginning immediately after fertilization to those that began during gastrulation. *Xenopus* embryos were exposed to ZD7288 (100 µM) from stage 1 through stage 10 or starting at stage 10 until stage 40, followed by anatomical laterality analysis at stage 45 (swimming tadpoles). Any instance of mirror image reversal of organ position, in the context of normal organ patterning and normal dorso-anterior index was counted as an instance of heterotaxia. Untreated embryos served as controls.

**Fig. 1.**
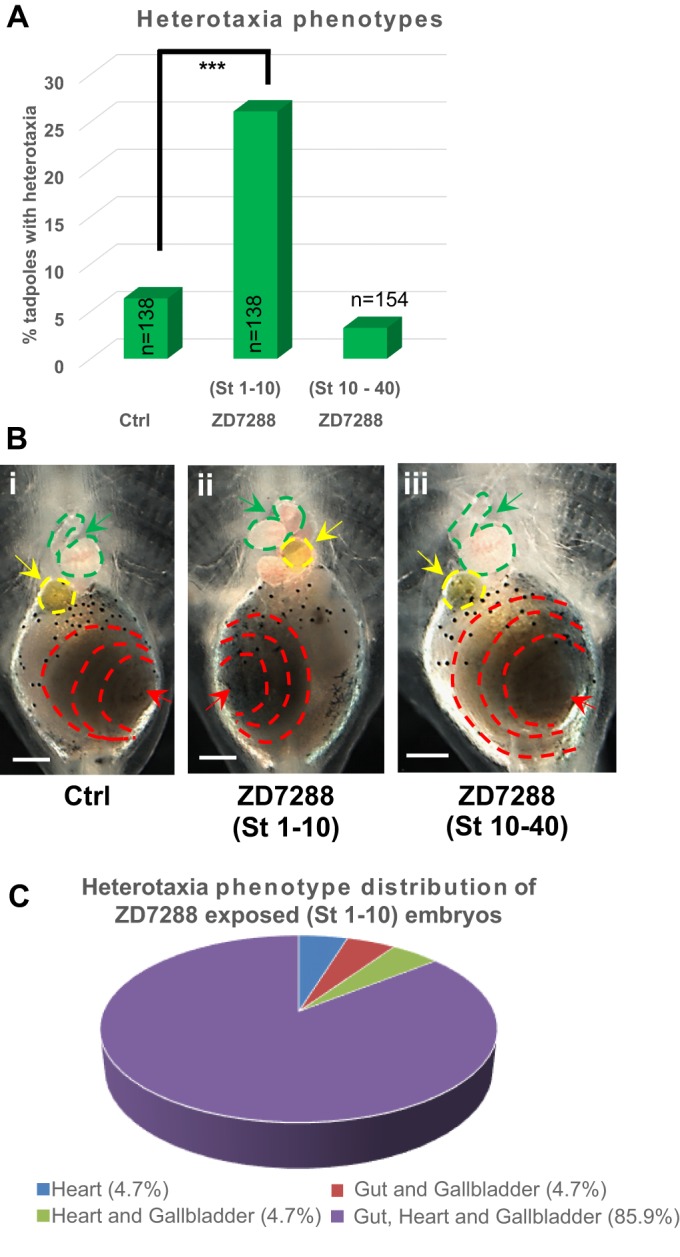
**HCN4 channel inhibitor ZD7288 affects left-right organ laterality only upon early exposure of embryos (St 1-10).** (A) Quantification of stage 45 tadpoles for left-right organ (heart, gut and gallbladder) laterality with or without exposure to 100 µM ZD7288 at 22°C at the indicated stages. A significantly high incidence of heterotaxia was observed in embryos exposed to ZD7288 between stages 1-10 in comparison to controls. Embryos exposed to ZD7288 late (St 10-40) did not show any significant increase in the incidence of left-right organ misplacement. The experiment was conducted in triplicates and data was pooled to run a χ^2^ analysis, ***P<0.001. (B) Representative images of stage 45 tadpoles: (i) Control tadpole showing rightward coiling gut as indicated by the red dotted lines and red arrow, rightward coiling heart as indicated by green dotted lines and green arrow, and leftward placed gallbladder as indicated by yellow dotted line and yellow arrow, (ii) tadpoles from embryos exposed to ZD7288 (100 µM – St 1-10) showing inversion of gut coiling as indicated by the red dotted lines and red arrow, inversion of the heart as indicated by green dotted line and green arrow, and inversion of gallbladder placement as indicated by yellow dotted lines and yellow arrow, (iii) tadpoles from embryos exposed to ZD7288 (100 µM – St 10-40) showing normal gut coiling as indicated by the red dotted lines and red arrow, normal heart as indicated by green dotted line and green arrow, and normal gallbladder placement as indicated by yellow dotted lines and yellow arrow. Scale bar: 0.25 mm. (C) Pie chart showing the incidence of various left-right phenotypes seen in the tadpoles from embryos exposed to ZD7288 (100 µM) between stages 1-10.

ZD7288 exposure during stages 1-10 induced a significantly high incidence (∼26%, *P*<0.001, χ^2^) of heterotaxia in comparison to controls (∼6%) (Fig. 1A,B). In sharp contrast, ZD7288 exposure during stages 10-40 did not induce heterotaxia (∼6%) in comparison to controls (∼6%) (Fig. 1A,B). Similar results were observed with another, more-specific HCN4 channel inhibitor ivabradine (Fig. S1). Distribution of heterotaxia outcomes from the early ZD7288 exposure showed various combinations of asymmetric placement of gut, heart and gallbladder (Fig. 1C). However, the majority (∼86%) of left-right asymmetric tadpoles showed *situs* *inversus* – full mirroring of all three organs (gut, heart and gallbladder) (Fig. 1B,C). Overall, percentage of ZD7288-treated tadpoles showing incorrect placement of each of the three organs were approximately equal (heart ∼34%, gut ∼32%, gallbladder ∼34%). Together, these results suggest that HCN4 function is required prior to embryonic stage 10 in establishing left-right asymmetry.

These studies took advantage of the temporal control afforded by pharmacological experiments beginning at different timepoints. Treatment with ZD7288, which inhibits HCN (*I_h_* currents), resulted in high incidence of *situs inversus*. These data implicate the HCN4 channel in early (prior to stage 10) processes involved in determination of left-right patterning in the *Xenopus* embryo.

### HCN4-DN (dominant-negative) causes heterotaxia

**Fig. 2.**
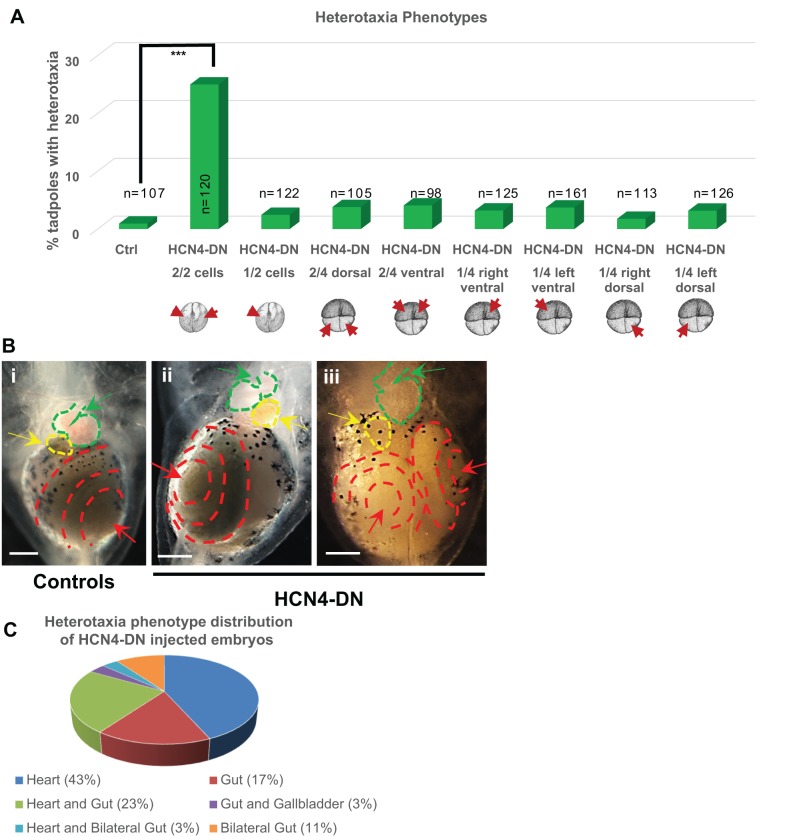
**Early injection of HCN4-DN affects left-right organ laterality in *Xenopus laevis*.** (A) Quantification of stage 45 tadpoles for left-right organ (heart, gut and gallbladder) laterality with or without microinjecting *HCN4-DN* mRNA apically (∼0.5-1 ng/injection/blastomere) in both blastomeres at 2-cell stage as indicated in the illustrations. A significantly high incidence of heterotaxia was observed in only when *HCN4-DN* mRNA was injected in both blastomeres at 2-cell stage, in comparison to controls. The experiment was conducted in triplicate and data was pooled to run a χ^2^ analysis, ***P<0.001. (B) Representative ventral images of stage 45 tadpoles: (i) Control tadpole showing rightward coiling gut as indicated by the red dotted lines and red arrow, rightward coiling heart as indicated by green dotted lines and green arrow, and leftward placed gallbladder as indicated by yellow dotted line and yellow arrow, (ii) *HCN4-DN* mRNA injected (both blastomeres at 2-cell stage) tadpoles showing inversion of gut coiling as indicated by the red dotted lines and red arrow, inversion of the heart as indicated by green dotted line and green arrow, and inversion of gallbladder placement as indicated by yellow dotted lines and yellow arrow, (iii) *HCN4-DN* mRNA injected (both blastomeres at 2-cell stage) tadpoles showing bilateral gut coiling as indicated by the red dotted lines and red arrow, with normal heart (green dotted lines and green arrow) and gallbladder (yellow dotted line and yellow arrow). Scale bar: 0.25 mm. (C) Pie chart showing the incidence of various left-right phenotypes seen in the *HCN4-DN* mRNA injected (in both blastomeres at 2-cell stage) tadpoles.

To molecularly validate whether HCN4 channels are specifically involved in organizing left-right organ laterality, we used an *HCN4-DN* (hyperpolarization-activated cyclic nucleotide-gated channel 4-dominant negative) mRNA construct that has been previously molecularly characterized and shown to inhibit the HCN4 channel current in mammalian cell culture by direct electrophysiology (Pitcairn et al., 2017). We confirmed that expression of HCN4-DN protein blocks HCN4 channel function and depolarizes the membrane voltage in *Xenopus* embryos (Fig. S2). We used a dominant-negative construct because many channels are present in *Xenopus* as maternal proteins (Adams et al., 2006; Aw et al., 2008, 2010; Levin et al., 2002; Morokuma et al., 2008; Qiu et al., 2005), and thus cannot be targeted by morpholinos or RNAi. *Xenopus* embryos were injected with *HCN4-DN* mRNA at 2- and 4-cell stage, in various blastomeres as indicated in Fig. 2A (red arrows), followed by anatomical laterality analysis at stage 45 (swimming tadpoles). As with the pharmacological inhibitor experiments, any deviation from the normal laterality of the three organs, in the context of otherwise normal patterning, was counted as an instance of heterotaxia. Uninjected embryos served as controls [as neither water nor non-specific mRNA, e.g. *β–galactosidase*, injections affect left-right asymmetry endpoints (McDowell et al., 2016b)]. *HCN4-DN* mRNA-injected tadpoles showed a significantly high incidence (25%, *P*<0.001, χ^2^) of heterotaxia only when injected in both blastomeres at 2-cell stage in comparison to controls (1%) (Fig. 2A,B). Distribution of all the different combinations of the three organs among the heterotaxic population is shown in Fig. 2C. The majority of phenotypes were inverted gut (∼17%), inverted gut and heart (∼23%), and inverted heart (∼43%) (Fig. 2C), with a few cases (∼10%) of bilateral gut (Fig. 2B,C). Overall, in *HCN4-DN* mRNA-injected tadpoles, gut (44%) and heart (54%) were the most affected organs. In contrast to *HCN4-DN*, *HCN2-DN* mRNA-injected tadpoles showed no effect on laterality (Fig. S3). Taken together, these data validate, in a gene-specific manner, the loss-of-function pharmacological inhibitor data implicating a role for HCN4 in LR patterning.

We then probed the time-dependency of HCN4 activity in left-right patterning by injecting at subsequent cleavage stages. We found that, even when adjusted for identical amounts and distribution of mRNA, injections performed after the second cell cleavage did not result in any significant incidence of left-right organ laterality defects in comparison to controls (Fig. 2A). We conclude that HCN4 channel activity is involved in determining heart and visceral organ laterality; moreover, its activity must occur very early during cleavage stages, as *HCN4-DN* mRNA introduced even at 4-cell stage is no longer effective at randomizing the left-right axis.

### The HCN4 channel is present very early in *Xenopus* embryonic development

**Fig. 3.**
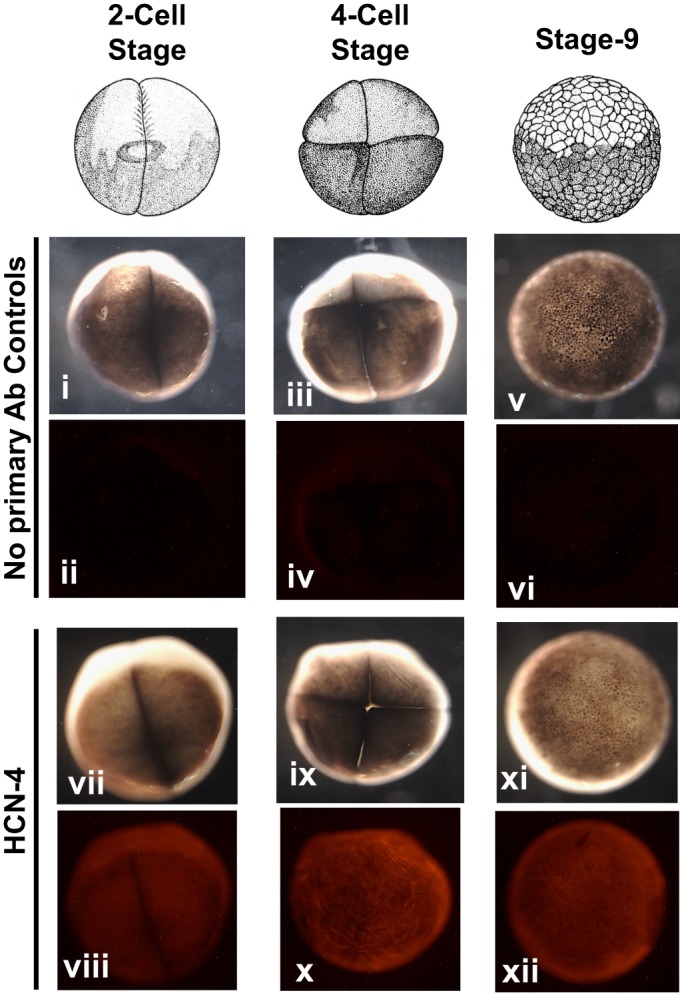
**Xenopus laevis embryos express endogenous HCN4 channel during early development.** Immunofluorescence analysis of whole *Xenopus* embryos for HCN4 channel protein at indicated stages of development. (i-vi) No primary antibody controls, (vii – xii) HCN4 immunofluorescence, (i, iii, v, vii, ix, xi) bright field images of immunofluorescent embryos, (ii, iv, vi, viii, x, xii) fluorescence images of immunofluorescence embryos. *Xenopus* embryos at the indicated stage of development showed a prominent HCN4 channel protein (*n*=15).

The presence and distribution of HCN4 channels during early embryonic developmental stages is not currently known. To assess this, we analyzed the spatio-temporal distribution of maternal HCN4 channels using whole embryo immunofluorescence on *Xenopus* embryos. HCN4 channel immunohistochemistry signal was detected as early as cleavage-stage (2-cell stage) and was present through stage 9 in *Xenopus* embryos (Fig. 3). No primary antibody-stained embryos were used as controls (Fig. 3). These results show that HCN4 channel is present very early 2-cell stage in developing frog embryos, and together with the bioelectric imaging and functional studies, are consistent with early exposure to HCN4-blocking reagents targeting endogenous physiological machinery operating at the earliest stages of LR patterning. Thus, we next sought to examine the transcriptional mechanisms known to be downstream of early bioelectric states.

### Neither HCN4-DN nor an HCN channel (*I_h_* current) inhibitor affect the sidedness of *Nodal* and *Lefty* transcriptional asymmetry

**Fig. 4.**
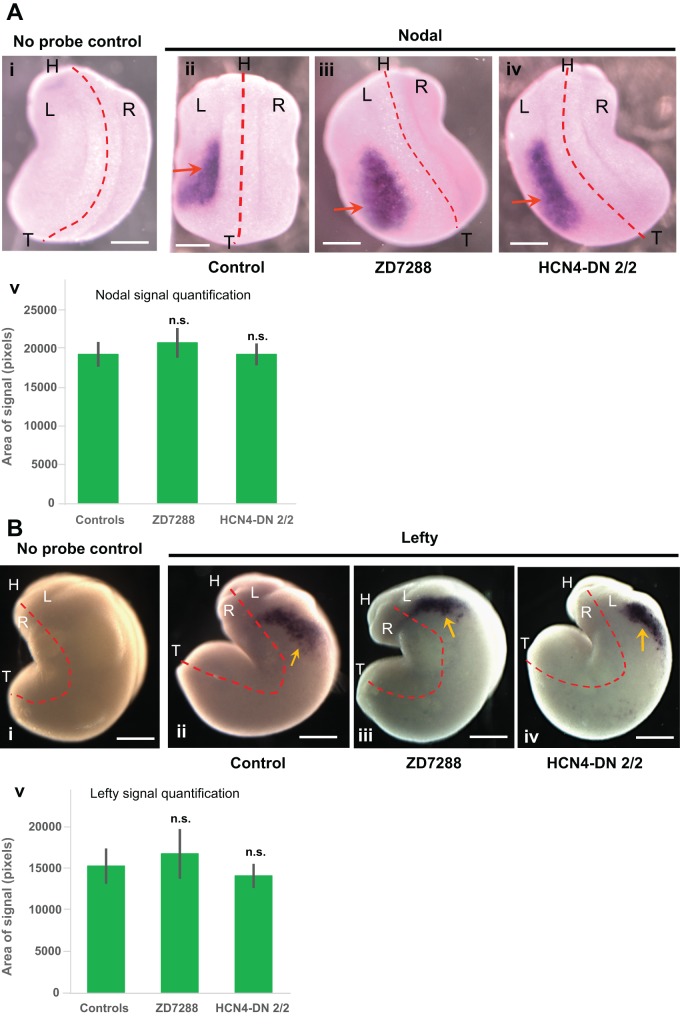
**Localization of the asymmetric gene Xnr-1 (nodal) is not affected by HCN4-DN and ZD7288.** (A) Representative images of approximately stage 21 embryos assayed for *Xnr-1 (nodal)* expression by *in situ* hybridization and quantification of area of *nodal* expression. Red dotted line is midline and L representing left-side, R representing right-side, H representing head and T representing tail of embryos. Scale bar: 0.25 mm. (i) No probe (negative) untreated control, (ii) control embryos with *Xnr-1* signal – red arrow, (iii) ZD7288-treated (from stage 1-10) embryos with *Xnr-1* signal – red arrow, (iv) *HCN4-DN* mRNA injected (in both blastomeres at 2-cell stage) embryos with *Xnr-1* signal – red arrows, and (v) quantification of area of *Nodal* expression in embryos showed no significant change in the area of *Nodal* expression in *HCN4-DN* mRNA-injected and ZD7288-treated embryos. *N*>10; data was analyzed by one-way ANOVA; n.s., non-significant. (B) Representative images of approximately stage 23 embryos assayed for *Lefty* expression by *in situ* hybridization and quantification of area of *lefty* expression. Red dotted line is midline and L representing left-side, R representing right-side, H representing head and T representing tail of embryos. Scale bar: 0.25 mm. (i) No probe (negative) untreated control, (ii) control embryos with *Lefty* signal – yellow arrow, (iii) ZD7288-treated (from stage 1-10) embryos with *Lefty* signal – yellow arrow, (iv) *HCN4-DN* mRNA-injected (in both blastomeres at 2-cell stage) embryos with *Lefty* signal – yellow arrows, and (v) quantification of area of *Lefty* expression in embryos showed no significant change in the area of *Lefty* expression in *HCN4-DN* mRNA-injected and ZD7288-treated embryos. *N*=10; data was analyzed by one-way ANOVA; n.s., non-significant.

The asymmetric position of the organs is regulated by a cascade of asymmetric gene expression (Levin, 2005; Ramsdell and Yost, 1998; Raya and Izpisua Belmonte, 2006); especially crucial are the left-side markers *Nodal*, *Lefty*, and *Pitx2*, and indeed the other known early mechanisms of asymmetry all act by randomizing the expression of these left-side determinant genes. Thus, we asked if HCN4 channel inhibition induces randomization by perturbing the normally consistent sidedness of this transcriptional cascade. To assess the epistasis between the *Nodal-Lefty-Pitx2* cassette and HCN4 function, we first performed whole embryo *in situ* hybridization with antisense probes against *Nodal* and *Lefty*. *Xenopus* embryos were either injected with *HCN4-DN* mRNA in both blastomeres at 2-cell stage (the misexpression condition that leads to heterotaxia, Fig. 2), or treated with the HCN channel (*I_h_* current) pharmacological inhibitor ZD7288 (100 µM) between stages 1-10. Uninjected and untreated embryos were used as controls. The embryos were fixed at stage 21 for *Nodal* (Fig. 4A, Table 1) and at stage 23 for *Lefty* (Fig. 4B, Table 2) *in situ* hybridization analysis.

**Table 1.**
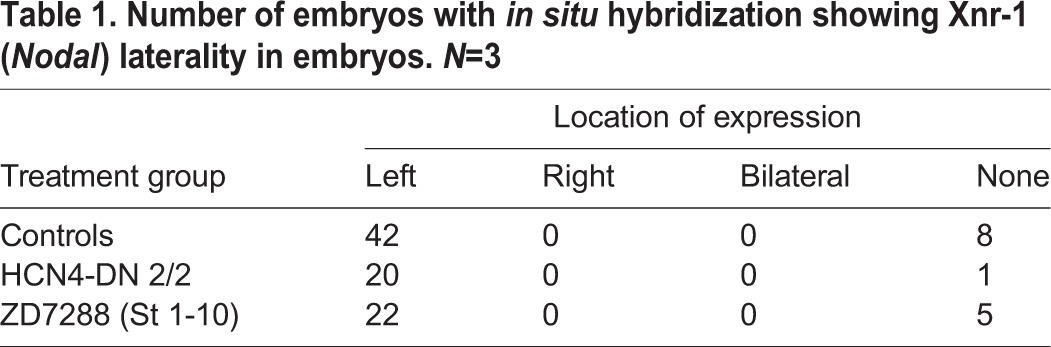
**Number of embryos with *in situ* hybridization showing Xnr-1 (*Nodal*) laterality in embryos. *N*=3**

**Table 2.**
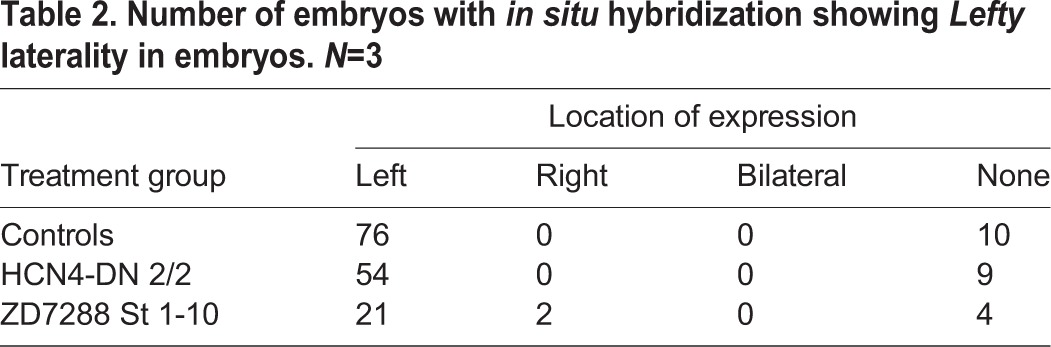
**Number of embryos with *in situ* hybridization showing *Lefty* laterality in embryos. *N*=3**

In controls, as expected, *Nodal* was expressed on the left side of the embryo in the majority (∼84%) of the embryos (Fig. 4A, Table 1). *HCN4-DN* mRNA*-*injected and ZD7288-treated embryos showed no significant change in the laterality of *Nodal* signal, with the majority of embryos (∼95% and ∼81%, respectively) expressing *Nodal* on the left side (Table 1). Quantification of area of *Nodal* expression domain showed no significant difference between the *HCN4-DN* mRNA*-*injected and ZD7288-treated embryos, and control embryos (Fig. 4Av, ANOVA). A subset of control and treated embryos from the same batch used for *in situ* analysis were raised to stage 45 and scored for left-right organ placement, confirming that *HCN4-DN mRNA* and ZD7288 were still effectively inducing heterotaxia in this cohort of animals, just as seen in Figs 1 and 2 (data not shown). We conclude that very early disruption of HCN4 function randomizes organ situs, bypassing the normal sequence of asymmetric *Nodal* expression.

In control embryos, *Lefty* was expressed on the left side in the majority (∼88%) of the embryos (Fig. 4B, Table 2). Strikingly, *HCN4-DN* mRNA*-*injected and ZD7288-treated embryos showed no significant change from wild-type embryos in the laterality of *Lefty* signal with majority of embryos (∼86% and ∼78%, respectively), showing *Lefty* on left side as in the set of control embryos (Table 2). Quantification of area of *Lefty* expression domain showed no significant difference between the *HCN4-DN* mRNA*-*injected and ZD7288-treated embryos, and control embryos (Fig. 4Bv, ANOVA). A subset of control and treated embryos from the same batch used for *in situ* analysis were raised until stage 45 and scored for left-right organ placement to verify that *HCN4-DN mRNA* and ZD7288 were still inducing heterotaxia in this cohort, as seen in Figs 1 and 2 (data not shown).

Thus, *HCN4-DN* mRNA and ZD7288 induce left-right organ randomization without altering the normal left-sided expression of *Nodal* and *Lefty*. These results reveal that HCN4 channel function affects organ placement, but bypasses the major asymmetry-regulating gene cassette, *Nodal-Lefty*.

### HCN4-DN and HCN channel (*I_h_* current) inhibition affect *Pitx2* expression

**Fig. 5.**
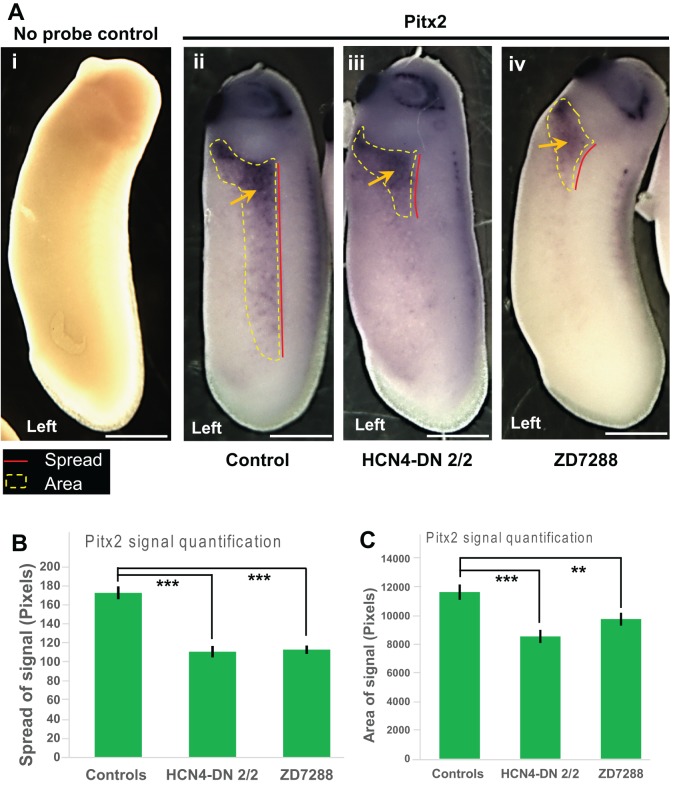
**Pitx2 expression is affected by HCN4-DN and ZD7288.** (A) Representative images of approximately stage 28 embryos assayed for *Pitx2* expression by *in situ* hybridization. Left orientation of the embryo is indicated at the bottom of the image. Red line indicates the anterior-posterior spread of the *Pitx2* expression and yellow dotted line indicates the area of the *Pitx2* expression. (i) No probe (negative) untreated control, (ii) control embryos with *Pitx2* signal – yellow arrow, (iii) embryos injected with *HCN4-DN* mRNA in both blastomeres at 2-cell stage with *Pitx2* expression - yellow arrow, (iv) ZD7288-treated (100 µM stage1-10) embryo with *Pitx2* expression - yellow arrow. Scale bar: 0.25 mm. (B) Quantification of anterior-posterior spread of *Pitx2* expression (as indicated by red lines in A) in embryos showed a significant reduction in the spread of *Pitx2* expression in *HCN4-DN* mRNA-injected and ZD7288-treated embryos. *N*=20; data was analyzed by one-way ANOVA; ***P<0.001. (C) Quantification of area of *Pitx2* expression (as indicated by yellow dotted lines in A) in embryos showed a significant reduction in the area of *Pitx2* expression in *HCN4-DN* mRNA-injected and ZD7288-treated embryos. *N*>25; data was analyzed by one-way ANOVA; ***P<0.001, **P<0.01.

To assess if HCN4 channel inhibition affects the normally left-sided expression of the late marker *Pitx2*, we performed whole embryo *in situ* hybridization against *Pitx2*. *Xenopus* embryos were either injected with *HCN4-DN* mRNA in both blastomeres at 2-cell stage or treated with a pharmacological inhibitor of HCN channels (*I_h_* currents), ZD7288 (100 µM), between stages 1-10. Uninjected and untreated embryos were used as controls [as these are known to be equivalent to water- or nonspecific mRNA-injected embryos (McDowell et al., 2016b)]. The embryos were fixed at stage 28 for *Pitx2* (Fig. 5, Table 3) *in situ* hybridization analysis. In controls, as expected, *Pitx2* is present on the left side of the embryo in majority (∼87%) of the embryos (Fig. 5A, Table 3). *HCN4-DN mRNA-*injected and ZD7288-treated embryos caused no significant change in the laterality of *Pitx2* signal, with a majority of embryos (∼81% and ∼87%, respectively) showing *Pitx2* on the left side of the embryos similar to controls (Table 3). A subsection of control and treated embryos from the same batch used for *in situ* analysis were raised to stage 45 and scored for left-right organ placement, confirming that *HCN4-DN* mRNA and ZD7288 were still inducing heterotaxia as seen in Figs 1 and 2 (data not shown). We conclude that the randomizing effects of HCN4 inhibition can bypass asymmetric *Pitx2* gene expression.

**Table 3.**
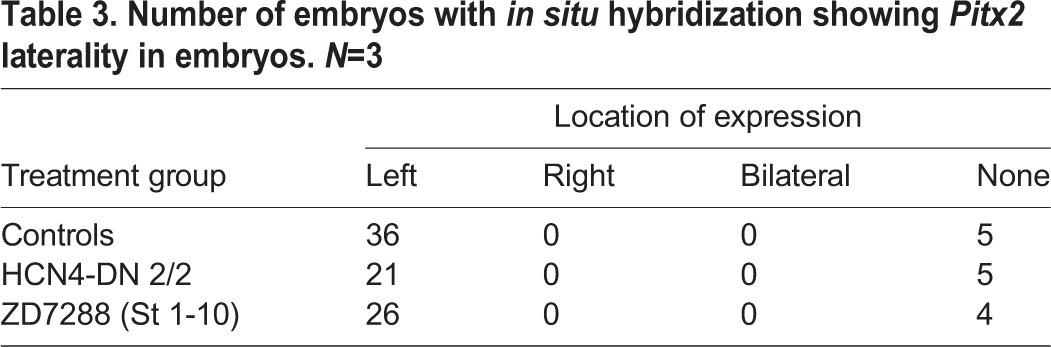
**Number of embryos with *in situ* hybridization showing *Pitx2* laterality in embryos. *N*=3**

Examining closely the results of treatments that randomized organs but not *Pitx2* situs, we observed that in contrast to the normal sidedness of expression, there was a significant difference in the spatial extent (pattern) of the *Pitx2* transcriptional domain in the treated embryos in comparison to controls. To analyze this, we quantified the anterior to posterior spread distance and area of the *Pitx2* signal in embryos (Fig. 5A-C). The anterior-posterior spread of *Pitx2* signal was significantly reduced (***P<0.001, ANOVA) in *HCN4-DN* mRNA*-*injected and ZD7288-treated embryos (Table 3). Similarly, the total area of *Pitx2* signal was significantly reduced (***P<0.001, **P<0.01, ANOVA) in *HCN4-DN* mRNA-injected and ZD7288-treated embryos (Fig. 5C). A similar analysis of the *Nodal* and *Lefty* expression domains revealed no significant changes in the signal pattern (Fig. 4). These results show that while *HCN4-DN* mRNA and ZD7288 treatments do not affect the laterality of *Pitx2* gene expression, both treatments significantly reduce the anterior to posterior spread distance and the area of *Pitx2* expression, while inducing left-right organ patterning defects in tadpoles.

## DISCUSSION

Here, we show that HCN4 channels are present in *Xenopus* embryos from the earliest stages of development, and play an important role in establishing left-right organ situs in *Xenopus* tadpoles. Blocking HCN4 channels results in heterotaxia and incorrect organ situs in tadpoles. This is effective only during early embryogenesis (pre-stage 10) as post stage 10, HCN4 channel blocking has no effect on organ situs. Even within the first 10 stages of embryogenesis, the HCN4-DN construct induces heterotaxia only when introduced in all blastomeres during early cleavage stages, further suggesting a very early role of HCN4 in establishing left-right asymmetry. Interestingly, an early block of HCN4 with ZD7288 and HCN4-DN does not have a significant effect on the sidedness of *Nodal-Lefty-Pitx2* gene expression, even though it leads to incorrect organ situs, suggesting it is acting either via a canonical pathway but bypassing *Nodal* and *Lefty* and directly affecting downstream targets or an alternative pathway of left-right asymmetry that is able to bypass the *Nodal-Lefty-Pitx2* cassette completely. Blocking of HCN4 channels (both pharmacologically and physiologically) induces a significant decrease in *Pitx2* signal in its anterior-posterior spread and area of the signal.

### Pleotropic actions of HCN4 in *Xenopus* embryonic development

Our recent study explored HCN4 function in embryonic cardiac tissue development (Pitcairn et al., 2017). That study and the experiments presented here used drastically different injection and culture conditions to target two different lineages/tissues: the animal cap versus the mesodermal heart lineage. The major differences were as follows. (1) In Pitcairn et al. (2017), where the role of HCN4 during heart development was the focus of study, the HCN4-DN was injected medially (along the equitorial plane of the embryo) which targets mostly mesodermal cardiac and kidney tissues. Here our injections were apical, to achieve a more global expression in the embryo, especially targeting the animal cap ectoderm and derived structures. (2) In Pitcairn et al. (2017), embryos were injected (equatorially) in one blastomere at the two-cell stage and kept in relatively high salt (1× MMR) until stage 9 (gastrulation). Under these high salt conditions, the ion gradients (and hence ion flux) for K^+^ and Na^+^ are reversed, affecting all K^+^ and Na^+^ ion channel fluxes: blocking HCN4 channel using HCN4-DN is a hyperpolarizing treatment and leads to subsequent incorrect *Nodal-Lefty-Pitx2* expression and gross morphology defects of heart. In contrast, the experiments reported here made use of injections (apical) into both blastomeres at the 2-cell stage, and the embryos were kept in low salt 0.1× MMR (mimicking its natural environment) throughout development. Under these conditions, an HCN4-DN-mediated block of HCN4 ion flux leads to depolarization (Fig. S2) with normal sidedness and pattern of *Nodal-Lefty-Pitx2* expression (Figs 4 and 5, Tables 1-3), but randomized organ situs (with normal organ morphology) (Figs 1 and 2).

The two different conditions help tease apart two different functions of HCN4: an early activity involved in establishing laterality of organs and another in developmental morphology of heart. Previously, it has been shown that moving the membrane voltage in either direction away from wild type leads to defects (Pai et al., 2012, 2015). Since early embryos already have quite depolarized membrane potentials, the HCN4-DN-mediated depolarization perturbation reported here may be too subtle to affect the asymmetry of the *Nodal-Lefty-Pitx2* expression, but is sufficient to affect a parallel pathway that feeds into organ situs. However, injections targeted to the developing heart in high salt conditions, combined with HCN4 blockade, induce a strong hyperpolarization perturbation (Pitcairn et al., 2017), which is a more drastic change that affects several gene regulatory networks involved in organ morphology, including feedback loops between *Pitx2* and HCN4 during cardiac morphgenesis (Christoffels et al., 2010; Clauss and Kaab, 2011; Wang et al., 2010). Work is currently ongoing in our laboratories to construct a detailed, testable, physiologically-realistic, and spatialized model of the early frog embryo's bioelectric circuits, chemical gradients, and relevant gene-regulatory circuitry (Pietak and Levin, 2016).

### HCN4 channels can act in a *Nodal-Lefty* asymmetric gene expression-independent manner

A recent series of studies and meta-analyses looking at the relation between causes of left-right abnormality, asymmetric gene expression of *Nodal-Lefty-Pitx2*, and organ situs, strongly suggested non-linearity of the laterality pathway (McDowell et al., 2016a,b; Vandenberg, 2012). Methods leading to disruption of asymmetric *Nodal* expression can still show correct organ situs, suggesting the presence of flexible gene regulatory networks and the presence of alternative pathways conferring redundancy and robustness to laterality and particularly organ situs. Some laterality defects were corrected even past *Pitx2* expression. Perturbation of early cytoskeleton, motor proteins, gap-junctions, and serotonin signaling, all showed significant repair [high incidence of mispatterned *Nodal*, but a lower incidence of organ situs defects (McDowell et al., 2016a)] (Fig. 3). However, a particularly interesting observation was that defects induced by perturbing laterality-relevant ion fluxes did not correct over time (incidence of organ situs defects were higher than incidence of incorrect sidedness of *Nodal*), strongly suggesting possible bioelectrically mediated *Nodal*-independent pathway that could correct for errors in the normal laterality-establishing transcriptional pathway.

Here we observed the opposite phenomenon: correct expression of asymmetric genes *Nodal and Lefty* but incorrect organ situs after HCN4 inhibition (Figs 1, 2, 4 and 5). In case of *Pitx2* the sidedness of expression was correct (left side) but the area of expression was significantly reduced. Although *Nodal* and *Lefty* expression was unaltered, it is possible that HCN4 inhibition interferes with the function of Nodal and Lefty proteins, resulting in decreased *Pitx2* expression and/or affecting other downstream pathways involved in left-right determination, thus acting through the pathway but bypassing the asymmetric gene expression of *nodal* and *lefty* genes. Alternatively, the decreased *Pitx2* gene expression may be due to interference in its intronic enhancer binding function. Although *Pitx2* is induced by *Nodal* (which is transiently expressed), its expression is maintained long after *Nodal* by binding of its intronic enhancer ASE with enhancers, including Nkx2 and Foxh1 (Shiratori et al., 2001, 2006). It is possible that HCN4 inhibition may interfere with this enhancer binding, leading to decreased *Pitx2* gene expression.

Previous studies have shown that *Pitx2* plays two important roles during organogenesis; the left-sided expression in the lateral plate mesoderm is important for overall embryonic left-right determination, while the asymmetric (left side) expression within individual organ's tissues (particularly gut and heart) guides asymmetric morphogenesis of these organs (Campione et al., 1999; Davis et al., 2017). Studies (both in mouse and *Xenopus*) (Clauss and Kaab, 2011; Davis et al., 2017; Poelmann et al., 2008) have shown that eliminating asymmetric *Pitx2* expression within organs like heart and gut disrupts asymmetric organ morphogenesis and also leads to disrupted organ morphologies. In our studies, the reduced (but correctly-sided) *Pitx2* signal in the lateral plate mesoderm may be affecting the *Pitx2* expression in the heart and gut organs. However, unlike the heart and gut *Pitx2* expression studies, we do not see morphological abnormalities in organ patterning. The gut and heart are placed in (left-right orientation) either normal or revered orientation but with fully formed morphologies.

**Fig. 6.**
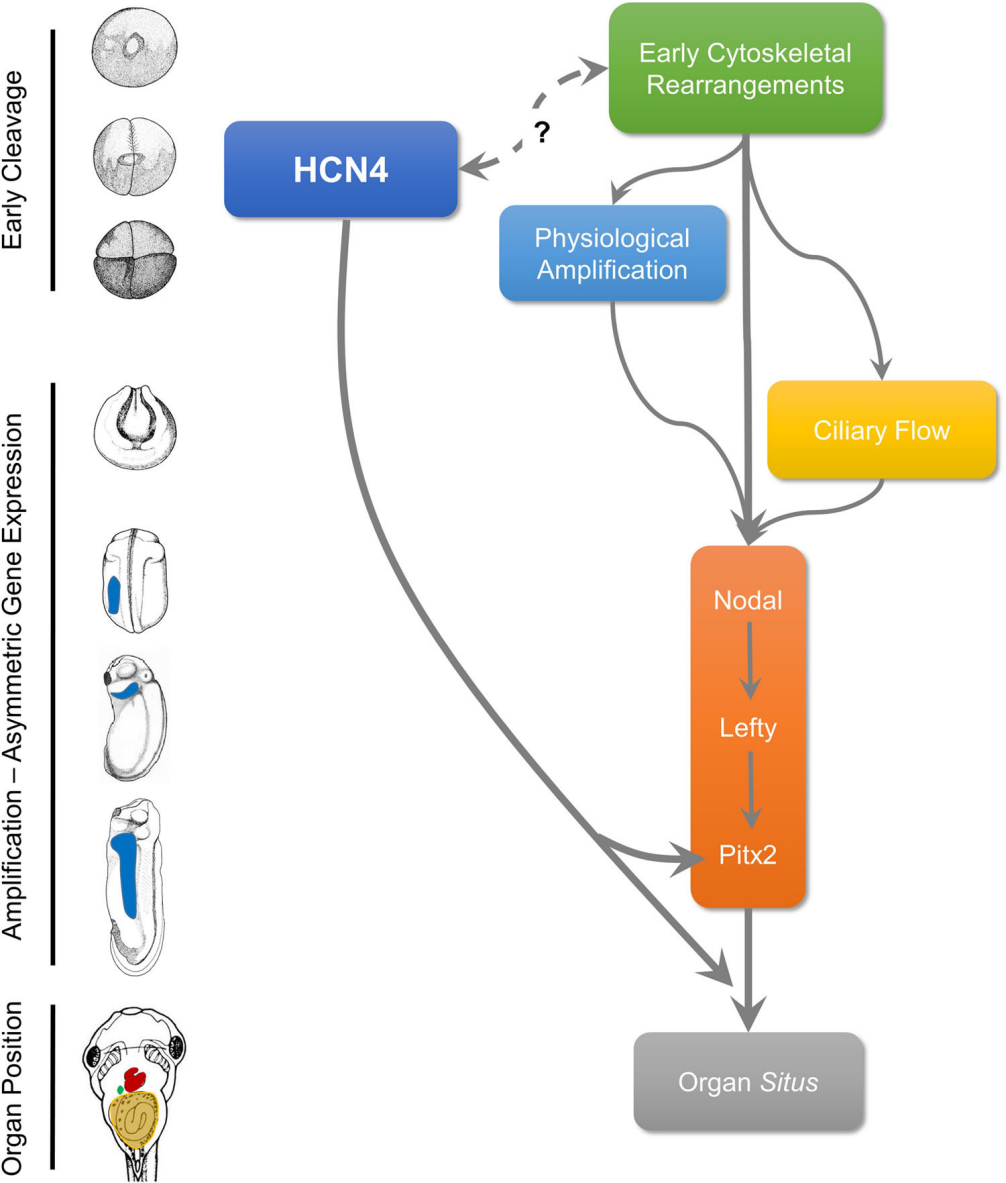
**Model for HCN4 function in establishing laterality.** The developmental timeline along the left illustrates early cleavage stages to post-gastrulation asymmetric gene expression of the *Nodal-Lefty-Pitx2* cascade leading to final organ situs. Previously established important laterality events are outlined adjacent to the developmental time line and portray very early laterality events of cytoskeletal rearrangement and physiological amplification, as well as later events such as gastrulation-stage ciliary flows, all funnel into the canonical *Nodal-Lefty-Pitx2* gene regulatory network to bring about invariant asymmetric organ situs. HCN4 action is required during early cleavage stages and can largely bypass the *Nodal-Lefty* asymmetric gene expression cascade to affect organ situs. HCN4-mediated *Nodal-Lefty* asymmetric gene expression-independent effect could be due to directly acting on downstream factors of the *Nodal-Lefty* pathway or through a non-canonical pathway. Players in this HCN4-mediated laterality patterning remain to be discovered.

Taken together, these data suggest that HCN4 channel-mediated ion flux as part of the pathways that bypass *Nodal-Lefty* asymmetric gene expression (Fig. 6). To our knowledge, there is only one other report of such a reagent: the Mahogunin (ubiquitin ligase) mutant (Mgrn1-C314D) causes left-right perturbation that bypasses asymmetric *Nodal* gene expression in mouse (Cota et al., 2006) and bypasses *Nodal-Lefty-Pitx2* axis asymmetric gene expression in *Xenopus* (McDowell et al., 2016b). It is not yet known whether Mahogunin and HCN4 are part of the same or different alternative pathways conferring robustness to laterality. It is also not yet known whether HCN4-mediated laterality pathways are present in other animals.

### HCN4 channel: downstream consequences of its inhibition

*Situs inversus* is the most frequent phenotype observed upon pharmacologically blocking HCN4 during early embryogenesis (Fig. 1C). In contrast, *HCN4-DN* mRNA injection-mediated HCN4 block (Fig. 2C) resulted in a wider spectrum of defects. This difference is most likely because injected *HCN4-DN* mRNA persists long beyond stage 9 and this effect on organ morphology may be due to HCN4-DN action in laterality overlapping with later organ morphogenesis steps (Pitcairn et al., 2017).

Left-right asymmetry establishment can be broadly categorized into three steps: (1) symmetry breaking, (2) orientation of the axes, and (3) amplification steps (Levin, 2005, 2006; Vandenberg et al., 2011). Disrupting each of these three steps is specifically predicted to have three different outcomes on organ situs. Disrupting the first step of symmetry breaking (cytoskeletal chirality, the directional action of motor proteins and transport) will cause the right and left halves of the embryos to be mirror-images of each other (*isomerism*), often observed in mice but rarely observed in *Xenopus* (Levin, 2005, 2006; Vandenberg et al., 2011). Disrupting the second step of the orientation of left-right axis with respect to anterior-posterior and dorso-ventral axis (which include asymmetric ion translocators and ion fluxes) will lead to a majority of individuals with complete reversal of asymmetric organs (*situs inversus*), as the left-right axis is randomly oriented with respect to the anterior-posterior and dorso-ventral axis (Levin, 2005, 2006; Vandenberg et al., 2011). Lastly, disruption of the third amplification step (which includes gap-junction communication, serotonin transport, ciliary flow, and *Nodal-lefty-Pitx2* cascade) will result in each organ making an independent decision leading to predominantly heterotaxic individuals.

Interestingly, blocking the HCN4 channel early leads to a major incidence of *situs inversus* and a small incidence of the bilateral gut (*isomerism*) (Figs 1 and 2); it should be noted that gut isomerism is extremely rare in the extensive *Xenopus* literature on left-right-randomizing treatments. This is consistent with HCN4 acting at the level of the second step of orientation of left-right axis in relation to the other two (anterior-posterior and dorso-ventral axes). This is further supported by the observation that HCN4 channels are present from early cleavage stages, and their role in laterality seems to be executed during early cleavage stages. Similar to HCN4, disrupting cytoskeletal dynamics causes a major incidence of *situs inversus* (Vandenberg et al., 2011). It is not yet clear whether cytoskeletal dynamics are upstream of HCN4 or if they are part of two independent mechanisms. The later is likely the case as we do not see consistent asymmetry in the distribution of HCN4 channel within the early embryo. What then could be upstream of HCN4 that is causing them to act physiologically in a different manner? A recent landmark set of studies showed that, as early as 8-cell stage, the left and right blastomeres are metabolically very different (Onjiko et al., 2016). It is possible that metabolic differences among blastomeres at the 2-cell stage lead to different physiology of HCN4 channel function.

### The timing and action of HCN4 channels in development: a very early role

Here we have identified a novel role of HCN4 channels during embryonic left-right patterning. Many previous reports indicate a critical role of ion translocators and ion fluxes in determining embryonic left-right asymmetry (Adams et al., 2006; Aw et al., 2008, 2010; Levin et al., 2002; Morokuma et al., 2008). In particular, asymmetric functions of H^+^/K^+^-ATPase and V-ATPase (Hibino et al., 2006; Kawakami et al., 2005; Levin et al., 2002; Shimeld and Levin, 2006), as well as two other potassium channels (KCNQ1 and K_ATP_) have been implicated. For the majority of these ion translocators, their asymmetric localization and action is post 4-cell stage, but before gastrulation. Crucially, HCN4 inhibition affects asymmetry during early embryogenesis – an observation that is incompatible with potential hypotheses about roles in regulating much later events like ciliary motion at gastrulation (Basu and Brueckner, 2008) since all of those events would be targeted by inhibitor exposure starting at stage 10. The same is true of many other highly-conserved elements of the left-right symmetry breaking machinery, such as cytoskeletal proteins (Davison et al., 2016; Lobikin et al., 2012), and reinforces the importance of focusing on intracellular, biophysical events as the earliest components of left-right pattering.

We found that HCN4 channels are already present in 2-cell embryos (most likely maternally loaded) and are uniformly expressed throughout the embryo all the way through gastrulation (Fig. 3). Hence, the action of HCN4 in establishing laterality is most likely at the physiological (post-translational gating) level of its function. This is not unprecedented, as in zebrafish the H^+^/K^+^-ATPase is uniformly expressed throughout the embryo and still is involved in laterality determination by its actions at the physiological level (Kawakami et al., 2005). Moreover, HCN4 is gated by a number of ligands (e.g. cAMP) which can be differentially localized to result in differential bioelectrical activity even if HCN4 protein is ubiquitous. Future studies using fluorescent reporters of cAMP and individual ion concentrations (being developed by a number of groups but not yet available in *Xenopus*), as well as transgenic *Xenopus* in which native HCN4 is labeled with a fluorescent tag, will dissect the very early steps of HCN4 activity. New techniques for introducing material into *Xenopus* eggs prior to fertilization may also be useful in manipulating this process, since injections even at 1-cell stage may be attenuated in their effects by the amount of time needed to make protein from the mRNA introduced then.

### Conclusion

Establishing invariant laterality is a fundamental aspect of most life forms across the tree of life. It is becoming clear that the mechanisms of establishing and executing laterality are redundant and highly robust to ensure correct organ situs even in presence of certain errors in the pathway. Many fascinating questions remain about the physiological processes that transmit and amplify physical chirality of intracellular cytoskeletal structures into embryo-wide programs of gene expression, and ultimately to the consistent asymmetry of organogenesis. The characterization of a novel player, the HCN4 channel, provides a new entry point into pathways which act very early during embryogenesis and then bypass the canonical asymmetric gene expression cascade of *nodal-lefty-pitx2* to exert their effects much later. Moreover, the discovery of new ion channels that underlie the endogenous bioelectric signaling that is increasingly seen to be an important component of developmental (Bates, 2015; Levin, 2012; Levin and Stevenson, 2012) and regenerative (Chifflet et al., 2005; Levin, 2014b; Wang and Zhao, 2010) patterning, adds to the toolbox of available targets for understanding and control of growth and form. The investigation of the dynamic interplay between early bioelectrics, subsequent transcriptional regulation, and resultant anatomical patterning presents exciting opportunities for understanding developmental and evolutionary dynamics. It is also possible that the study of compensatory redundant pathways will reveal new approaches for harnessing the robustness of developmental mechanisms for regenerative medicine.

## MATERIALS AND METHODS

### Animal husbandry

*Xenopus laevis* embryos were fertilized *in vitro* according to standard protocols (Sive et al., 2000) in 0.1× Marc's Modified Ringer's (MMR; 10 mM Na^+^, 0.2 mM K^+^, 10.5 mM Cl^−^, 0.2 mM Ca^2+^, pH 7.8). *Xenopus* embryos were housed at 14-18°C (14°C overnight after injection and subsequently at 18°C), except during drug exposure which was at 22°C, and staged according to Nieuwkoop and Faber (1967). All experiments were approved by the Tufts University Animal Research Committee (M2014-79) in accordance with the guide for care and use of laboratory animals.

### Microinjections

Capped synthetic mRNAs generated using mMessage mMachine kit (Ambion) were dissolved in nuclease free water and injected into embryos immersed in 3% Ficoll using standard methods (Sive et al., 2000). Each injection delivered between ∼0.5-1 nl (0.5-1 ng) of mRNA (per blastomere) into the embryos, at the indicated stages into the middle of the cell in the animal pole. *HCN4-DN* was a mammalian (mouse) HCN4, modified as per Pitcairn et al. (2017). Briefly, a standard approach was used for generating dominant-negative channel subunit (Kuzhikandathil and Oxford, 2000; Preisig-Muller et al., 2002; Xue et al., 2002). The HCN4 channel function was abolished by altering the highly conserved cation selective sequence in the pore domain (changed from GYG_349-351_ to AAA_349-351_) to generate HCN4-(AAA)-DN mutant from HCN4-WT using primers: 5′CACATGCTGTGCATTGAGGACGAACGTCAGGCA-3′ (forward) and 5'-TGCCTGACGTTCGTCCTCAATGCACAGCATGTG-3′ (reverse). The construct was subcloned into a pCS2 vector to transcribe into mRNAs for *Xenopus* microinjections.

### Anatomical laterality assays

*Xenopus* embryos were analyzed as in Levin and Mercola (1998) for position (situs) of three organs: heart, gut and gallbladder at stage 45 (Nieuwkoop and Faber, 1967) using fiber light illumination from the ventral side. Heterotaxic embryos were defined as ones having a reversal in one or more organs. Treatments were titered to levels that gave rise to >90% embryos with normal dorso-anterior development and correctly-formed organs. All reported left-right inversions are embryos with clear (unambiguous) left-right organ situs. It is important to note that our analysis is extremely stringent – it underestimates the overall effect of any given treatment. Even if 100% of embryos are affected by some manipulation, the maximum observable effect will still be capped at 87.5% as some percentage of embryos will have all three organs randomly land in correct orientation making them indistinguishable from the wild type and hence will be scored as normal.

### Drug exposure

*Xenopus* embryos were incubated in pharmacological blocker of HCN4 channel ZD7288 (Tocris biosciences) (100 mM stock solution in water) dissolved in 0.1× MMR (final concentration 100 µM) during the stages indicated in respective experiments followed by several washes with 0.1× MMR. Note that these experiments were performed at 22°C since ZD7288 effect on embryonic left-right asymmetry was found stronger at 22°C and not at 14°C and 18°C (data not shown) (Yanagida et al., 2000). Untreated embryos reared at 22°C served as controls.

### Imaging V_mem_ using CC2-DMPE: DiBAC_4_(3)

CC2-DMPE and DiBAC_4_(3) voltage reporter dyes were obtained from Invitrogen and used as per the standard protocol, including dark-field and flat-field correction (Adams and Levin, 2012). Briefly, the use of two dyes with opposite emission profiles simultaneously provides an internal control and allows ratiometric normalization. CC2-DMPE stock (5 mM) was dissolved 1:1000 in 0.1× MMR and the embryos were incubated in dark in this solution for at least 1 h followed by washes with 0.1× MMR. DiBAC_4_(3) stock (1.9 mM) was dissolved 1:4000 in 0.1× MMR and the CC2-DMPE-stained embryos were then incubated in dark in this solution for at least 30 min followed by visualization under the microscope. An Olympus BX-61 microscope equipped with a Hamamatsu ORCA AG CCD camera, and controlled by Metamorph software (Molecular Devices), was used to collect signal. NIH Image J software was used to quantify the fluorescence intensities of the CC2-DMPE:DiBAC signal.

### Intracellular recordings from embryo cells

Membrane potentials were measured using an oocyte clamp OC-725C amplifier (Warner Instruments, Hamden, CT, USA) with a single voltage electrode. Microelectrodes were made from thin-walled borosilicate glass pulled with a flaming/brown micropipette puller (p-97, Sutter Instruments, Novato, CA, USA) and back filled with electrode solution (2 M potassium acetate, 10 mM KCl, 5 mM HEPES pH 7.5). Tip resistances were 80-100 MΩ. Electrode penetration of ectodermal cells was by visual guidance on a fixed-stage microscope (Zeiss) using a three-axis micromanipulator.

### Immunofluorescence

Spatial distribution of HCN4 channel in embryos was detected by immunofluorescence for the HCN4 channel on whole embryos. Briefly, embryos were fixed overnight in MEMFA at 4°C (Sive et al., 2000). The embryos were permeabilized in PBS 0.1%Triton-X-100, blocked with 10% goat serum in PBST for 1 h at room temperature, and incubated at 4°C overnight with primary antibody (Anti-HCN4 – rabbit polyclonal; Abcam ab66501) for HCN4 at 1:500 dilution in PBST+10% goat serum (blocking buffer). Embryos were washed six times in PBST and incubated with Alexa Fluor-conjugated fluorescent secondary antibody (Invitrogen) at 1:500 dilution in PBST+10% goat serum overnight at 4°C. Embryos were washed six times in PBST and photographed using Nikon SMZ-1500 scope with Q-capture software.

### *In situ* hybridization

*Xenopus* embryos were collected and fixed in MEMFA (Sive et al., 2000) and *in situ* hybridization was performed as previously described (Harland, 1991; Sive et al., 2000). The embryos were washed with phosphate buffered saline 0.1% Tween-20 (PBST) and transferred through series of methanol washes (25%, 50%, 75%, 100%). *In situ* anti-sense probes were generated *in vitro* from linearized templates using a DIG labeling mix (Roche). Chromogenic reaction times were optimized for signal to background ratio. Probes used were: *Xnodal* (Sampath et al., 1997), *Xlefty* (Meno et al., 1997), and *Xpitx2* (Campione et al., 1999). NIH Image J software was used to quantify the *in situ* signal.

### Statistics

All statistical analysis was performed using Microsoft Excel. As appropriate for each case, data were either pooled from multiple repeat experiments, with χ^2^ analysis performed on them, or data from various iterations was analyzed by *t-*test (for 2 groups) or ANOVA (for more than two groups), as indicated with each experiment.
